# Investigating an Emerging Virus During a Sudden Pandemic Outbreak

**DOI:** 10.5041/RMMJ.10414

**Published:** 2020-07-31

**Authors:** Ronit Sarid

**Affiliations:** The Mina and Everard Goodman Faculty of Life Sciences & Advanced Materials and Nanotechnology Institute, Bar Ilan University, Ramat-Gan, Israel

**Keywords:** Coronavirus, coronavirus disease 2019, human coronavirus, severe acute respiratory syndrome

## Abstract

At the time of writing, in July 2020, the recently emerging SARS-CoV-2 pandemic has attracted major attention to viral diseases, in particular coronaviruses. In spite of alarming molecular evidence, documentation of interspecies transmission in livestock, and the emergence of two new and relatively virulent human coronaviruses within a 10-year period, many gaps remain in the study and understanding of this family of viruses. This paper provides an overview of our knowledge regarding the coronavirus family, while highlighting their key biological properties in the context of our overall understanding of viral diseases.

## INTRODUCTION

In March 2015, Bill Gates, the cofounder of Microsoft, gave a TED presentation in which he stated that while many countries are investing in developing nuclear defense measures, they should be investing similar efforts in developing protection measures against deadly virus pandemics.^1^ He referred to the 2014 Ebola virus outbreak, which killed more than 11,000 people in West Africa, and warned that the USA and other countries were not ready for the future epidemic that could be even more devastating. “If anything kills over 10 million people in the next few decades, it’s most likely to be a highly infectious virus rather than a war,” Gates said. Gates repeatedly urged governments to prepare for pandemics in a manner similar to their preparations for war. Five years later, in 2020, when the World Health Organization declared the coronavirus disease 2019 (COVID-19)—caused by the newly emerging SARS-CoV-2 virus—to be a pandemic, this talk was widely acknowledged to be almost prophetic. Unfortunately, society, governments, public health officials, pharmaceutical companies, and researchers were not properly prepared for this outbreak.

Concerns about the potential emergence of an influenza virus pandemic have been raised and extensively discussed over many years. These concerns were based on the destructive 1918 Spanish pandemic flu, as well as on subsequent influenza virus outbreaks, the most recent of which emerged in Mexico in 2009.^2^ Little attention was paid to coronaviruses, which were temporarily in the spotlight when the severe acute respiratory syndrome (SARS) disease emerged in China in 2002,^3^–^5^ and later when the Middle East respiratory syndrome (MERS) appeared in Saudi Arabia in 2012.^6^,^7^ These outbreaks attracted some research interest to coronaviruses, yet large pharmaceutical companies did not devote substantial resources to these diseases, because therapies and vaccines for these diseases did not appear likely to be profitable. Nevertheless, a clear potential threat by coronaviruses was recognized by a number of investigators.^8^–^11^ This was based on the wide range of viral reservoirs and previous documentation of cross-species spread in veterinary medicine, as well as knowledge of their replication mechanism.

Our understanding of other viruses, in particular regarding coronaviruses, can provide guidance for understanding and control of the SARS-CoV-2 new outbreak. Yet, many gaps remain in the study and understanding of these viruses. This paper will discuss recent emerging evidence on the SARS-CoV-2 virus in the context of our general understanding of viral diseases.

## THE CORONAVIRUS SUBFAMILY—GENERAL FEATURES

COVID-19 is caused by a newly identified virus, SARS-CoV-2, belonging to the *Coronavirinae* subfamily in the *Coronaviridae* family. Based on phylogenetic relationship and genomic organization, this subfamily has four different genera—alpha, beta, gamma, and delta—of which alpha- and betacoronaviruses have mammalian hosts, while the reservoir of the gamma- and deltacoronaviruses is mainly birds. Sequencing of the SARS-CoV-2 genome was accomplished soon after its discovery, promoting its classification to the betacoronavirus and subsequent attribution of certain basic properties that can permit some basic assumptions.

Coronaviruses (CoVs) are the largest known RNA viruses, encoding 26–32 kb positive-sense single-stranded RNA with a 5′-terminal cap structure and a 3′-polyadenylated tail. Virions of coronaviruses have a mean diameter of 120 nm, while their most notable feature is a halo of spikes that project from the envelope and give the viral particle the shape of a solar corona, inspiring the name of this family of viruses. Four major structural proteins form the coronavirus particles: the nucleocapsid (N) phosphoprotein, which forms a helical ribonucleoprotein core together with the viral genome; the trimeric spike (S) glycoprotein, which mediates virion attachment to host cell receptor and membrane fusion; the membrane (M) protein, which is highly abundant and shapes the virion envelope; and the low-abundance integral envelope (E) protein, which plays an important role in viral envelope assembly. Certain coronaviruses also include the hemagglutinin-esterase (HE) protein, which forms short protrusions beneath the S protein spikes. All structural genes, except the HE, are encoded in the 3′-most third of the genome, while the other two-thirds encode the viral non-structural proteins (NSPs), such as replication enzymes and additional accessory genes. Two partially overlapping open reading frames, ORF1a and ORF1b, are encoded in this region, producing the ORF1a polypeptide or being continuously translated via ribosome frameshifting to produce a large ORF1ab polyprotein. These polyproteins are cleaved by virus-encoded proteinases into a number of mature NSPs. As cleavage of the polypeptide is essential for production of functional viral proteins, drugs that target these proteinases have potential therapeutic applications. The assembled NSP replicase–transcriptase complex (RTC) is responsible for RNA replication and transcription. Of note, the coronaviruses encode several viral proteins with RNA-modifying or replication activities, including RNA-dependent RNA-polymerase (RdRp), a helicase, an exonuclease (ExoN), and 2′-O-ribose-methyltransferase.^12^

Replication of the CoV RNA genome takes place on cellular membranes while generating dramatic changes in their organization. Replication involves the synthesis of a full-length negative-strand RNA, which serves as a template for the synthesis of additional positive-sense RNA genomes. The 5′-most open reading frames, ORF1a and ORF1ab, are translated from full-length genomic RNA, while a series of subgenomic (sg) mRNAs, which commonly include the 60–100-nucleotide untranslated leader sequence from the 5′ end, and extend to the 3′ end of the RNA genome, are produced to enable translation of downstream genes. Production of these mRNAs involves discontinuous transcription of the full-length positive-strand RNA genome. This process may encompass switching between templates and can therefore result in homologous or heterologous recombination when more than one CoV virion infects a given cell. This capacity, along with the high theoretical mutation rate of the RNA genome by RdRp (10^4^ nucleotide substitutions/site/ year) can potentially enable rapid genetic changes in coronaviruses. Accordingly, long-term coronavirus infection in some animals, combined with high mutation and recombination rates, increases the probability that a virus mutant with an extended host range might arise.^8^,^13^,^14^ Nevertheless, a proofreading function during CoV replication is provided by the viral protein ExoN and may balance between fidelity and misincorporation rate that could result in lethal mutagenesis. ExoN inhibitors are expected to reduce the fidelity of CoV replication and thus promote high-level mutagenesis of the viral genome that could drive virus extinction.^15^–^17^

## CORONAVIRUSES PATHOGENESIS

Before the 2002 SARS epidemics, CoVs were predominantly associated with devastating outbreaks of severe diseases in livestock, mostly gastroenteritis and bronchitis, affecting domestic animals including cattle, pigs, and chickens. Coronaviruses also infect a variety of wild animals including birds, snakes, mice, and bats.^8^ Human CoV (hCoV) infection (Table 1) was first described in the 1960s and was primarily associated with mild upper respiratory diseases (the “common cold”) caused by hCoV-229E and hCoV-OC43.^18^–^20^ Two other hCoVs were identified almost 40 years later: hCoV-HKU1, which was linked with chronic pulmonary disease,^21^ and hCoV-NL63, which was associated with both upper and lower respiratory tract disease in children and adults.^22^ In addition, hCoV-NL63 was associated with croup in infants and young children. Interestingly, association of hCoV-NL63 with Kawasaki disease, an inflammation of blood vessels that mostly occurs in children, has been reported,^23^ yet subsequently failed to be confirmed.^24^,^25^ All four reportedly mild pathogenic coronaviruses are associated with 10%–30% of cases of the common cold,^26^–^28^ yet they have the potential to cause severe lower respiratory tract infection in infants, in the elderly, and in patients with other underlying illness,^29^ while hCoV-OC43, like SARS-CoV-2, has been associated with neurologic dysfunction as well.^10^ Interestingly, sequence and phylogenetic analysis of hCoV-OC43 dates its transmission from cattle into the human population to around 1890, concurrently with a pandemic which killed approximately 1 million people worldwide and was traditionally ascribed to influenza virus.^10^ The three other hCoVs can infect the lower respiratory tract and cause a severe acute respiratory syndrome (SARS). The SARS-CoV was identified with the emergence in 2002 of an atypical pneumonia that resulted in a 10% mortality rate, and MERS-CoV subsequently emerged in 2012 and caused 30% mortality.

**Table 1:** Human Coronaviruses (hCoVs).

Species	Genus	Year of Discovery	Disease Association	Cellular Receptor
hCoV-229E	Alphacoronavirus	1966	Common cold, pneumonia	Human aminopeptidase N (APN)
hCoV-OC43	Betacoronavirus	1967	Common cold, pneumonia, neurological dysfunction	9-O-acetylated sialic acid
hCoV-SARS (SARS-CoV)	Betacoronavirus	2003	Severe-acute respiratory syndrome	Angiotensin-converting enzyme 2 (ACE2)
hCoV-NL63	Alphacoronavirus	2004	Common cold, pneumonia, bronchiolitis	Angiotensin-converting enzyme 2 (ACE2)
hCoV-HKU1	Betacoronavirus	2005	Common cold, pneumonia	9-O-acetylated sialic acid
hCoV-MERS (MERS-CoV)	Betacoronavirus	2012	Severe-acute respiratory syndrome	Dipeptidyl peptidase 4 (DPP4)
HCoV-SARS-2	Betacoronavirus	2020	Severe-acute respiratory syndrome	Angiotensin-converting enzyme 2 (ACE2)

Both hCoV-229E and hCoV-NL63 are alphacoronaviruses, while all other hCoVs belong to the betacoronavirus genus. All known hCoVs originated from animals, with rodents or bats being the most likely ancestors, and passed to humans either directly or through an intermediate host.^8^,^30^ Other examples of transmission across species barriers occurred with hCoV-OC43, which crossed species to infect dogs,^31^ and were suggested for SARS-CoV-2, which can infect ferrets and cats.^32^ The occurrence of pathogenic hCoV outbreaks with a zoonotic origin has led to the search for animal reservoirs and the identification of a multitude of previously unknown coronaviruses, mostly in birds and bats, that have the potential to cross species. This reinforces the need to monitor animals to gain control over the emergence of these viruses.

## HOST CELL ENTRY RECEPTOR

An important parameter towards understanding any newly emerging virus concerns virus particle attachment to host cells. Attachment determines virus host range and may help in understanding its pathogenesis. Attachment of CoVs to the host cell receptor is mediated by the surface transmembrane S glycoprotein, which may also induce cell-to-cell fusion. The S protein is synthesized as a single polypeptide that is cleaved by cellular proteases into two polypeptides, S1 and S2. This cleavage may take place during virion assembly and release from the infected cell or during initiation of infection. The S1 protein, composed of the S protein amino terminal domain, forms the tip of the spike, and contains the receptor-binding domain (RBD) of most CoVs. Of note, S was suggested to be a modular protein that can exchange domains and thus provides a low barrier for selection of new coronaviruses that were either generated by mutations or recombination. The host protein, angiotensin-converting enzyme 2 (ACE2) was found to function as a critical determinant for hCoV-NL63, SARS-CoV, MERS-CoV-2, and SARS-CoV-2 cellular tropism.^33^ Like other viral receptors ACE2 is a cellular molecule which has an important physiologic function. Decoding the molecular and structural bases for receptor usage is crucial for understanding cross-species transmission but may also suggest potential therapeutic strategies. Development of animal models for SARS-CoV-2 infection is vital in providing comprehensive understanding of the pathogenic mechanisms involved but may also serve for screening anti-viral drugs and vaccines.

## DIAGNOSTIC MEASURES

Accurate large-scale diagnostic testing is critically important in the control of a pandemic outbreak. It is especially important for a novel virus with no specific treatment and no vaccine. Identification of infection among hospitalized patients is of high priority to prevent nosocomial infection and subsequent community transmission. Ideal diagnostic tests are highly specific and sensitive, avoiding false-positive and false-negative outcomes, respectively.

Viral infections can be diagnosed through the detection of viral antigens or genomes by various methods, of which the most widely used is based on amplification of viral genomic sequences. Indeed, SARS-CoV-2 testing is based on respiratory specimens, either oropharyngeal, nasopharyngeal, or both, that undergo RNA extraction followed by reverse-transcriptase polymerase chain reaction (RT-PCR). Several assays have been optimized to have high sensitivity and minimal cross-reactivity with circulating viruses present in the community. These tests can diagnose or rule out COVID-19 in suspected patients and may also track the infection in those who were in contact with infected individuals. Scaling up these tests to expand testing capacity has been a major goal during the COVID-19 outbreak. Other nucleic acid-based diagnostics, such as nucleic acid sequencing, were established and are commercially available. Of note, inadequate sample collection may yield false negative tests. Early implementation of SARS-CoV-2 diagnostics for the identification and subsequent isolation of infected individuals has been helpful in control of the outbreak. However, different implementation strategies and indications for diagnostic testing were chosen in different countries depending on public health policies, testing capacity, and the morbidity rate.^34^

An alternative to nucleic acid detection is to identify viral antigens, in particular those that are highly abundant, such as the SARS-CoV-2 nucleocapsid protein. Such assays, which are generally rapid and based on immunoassay, are under development for SARS-CoV-2.

Finally, serologic tests to identify a virus-specific immune response, including IgM and IgG antibodies, have potential diagnostic implications. The antibody response to infection takes days to weeks to be detectable. Therefore, these tests cannot be used to detect an acute infection and will generally also be negative during the first days post infection. However, accurate serologic assays enable rapid screening and evaluation of the proportion of infected individuals within a given population, herd immunity, and epidemiologic assessments. Furthermore, quantitative serologic assays combined with virus neutralization assays could help define the long-term effectiveness of the anti-viral immune response. Several immunoassays have been developed for SARS-CoV-2, though their accuracy remains to be fully validated.

## EPIDEMIOLOGY

Epidemiological investigations have played major roles in mitigating COVID-19. In the context of an emerging virus, epidemiologists gather data regarding new cases, including their timing, location, origin, and outcome. The data are based on diagnosed cases that present symptoms, as well as on laboratory-confirmed cases of infection, which could also be asymptomatic. Potential animal reservoirs and sources of environmental contamination are also being evaluated. The collected data are incorporated into models that illustrate modes of transmission, such as droplet infection, and predict the infection spread. Populations at high risk for infection and severe outcome, such as the elderly or individuals with chronic disease, can also be identified via epidemiologic investigation. Among other parameters, the prediction of disease spread is based on the basic reproductive number (*R0*) representing the average number of people who will become infected directly by a single infected individual. One of the goals of epidemic containment is to restrain the transmission of the virus and reduce *R0*, which is modulated by the time interval of viral shedding, viral infectiousness, and the interface that mediates transmission between infected and susceptible individuals. Understanding how a virus spreads in a population, together with knowledge on the virus stability in the environment (e.g. aerosol, water, or different surfaces), helps develop guidelines for prevention and containment. Sequence analysis of viral genomes may also help tracking virus dissemination within a population. Epidemiology also allows determination of case fatality rate (CFR), although it is very difficult to determine this value during an ongoing outbreak, in particular when the number of asymptomatic infections is high. Finally, research has shown that people become infectious before any symptoms of illness appear, during the pre-symptomatic incubation period, while others do not develop a disease and remain asymptomatic. Asymptomatic individuals may be those in whom the virus replication is low and therefore their ability to spread the virus is limited. However, because disease symptoms are affected by the immune response, lack of symptoms may reflect a well-balanced immune response and does not necessarily correlate with high or low viral loads. Either way, it is interesting to examine the relationship between the severity of the disease and the response of the innate, humoral and cellular immune systems over time. Knowing the proportion of asymptomatic infections and whether asymptomatic individuals are contagious and can spread the virus are top public health objectives which may help define ways to avoid infections, such as wearing masks and social distancing.

## ANTI-VIRAL TREATMENTS

Drug treatment for most viral diseases is limited, and relies primarily on symptomatic treatment and supportive care with no attempt to impair virus propagation. This approach rests on the notion that viruses are cellular parasites that employ cellular processes to reproduce, and hence any attempt to target their propagation is likely to be toxic to the host cells as well. Therefore, few anti-viral drugs are available with a sufficient level of safety. In addition, anti-viral drugs must be highly effective, as incomplete inhibition of the virus infection may enable selection of drug-resistant virus strains that can cause an even greater threat. Accordingly, most anti-viral compounds target viral enzymes, such as proteases^12^,^35^ and replication enzymes.^36^,^37^ New anti-viral drugs, some of which are still under development, focus on blocking attachment of the virus to the host cell, preventing its entry into cells and virion uncoating, inhibiting regulatory functions, or targeting the viral RNA.^38^ Potential drugs with such activities can be identified by using purified viral proteins in high-throughput mechanism-based experimental screens. Computational approaches, based on the atomic structure of the target molecule, enable virtual screening and design of new drugs. In addition, a comprehensive understanding of the molecular interactions between the virus and the host cell, together with whole-genome sequencing and methods to block gene expression, enables identification of cellular gene products that are essential for virus replication, and which can be used as targets for treatment without damaging the host. Obviously, any new compound has to reach appropriate bioavailability and pharmacokinetics with no toxic effects.

The success of developing a number of agents to treat or inhibit human immunodeficiency virus (HIV) infection, as well as anti-hepatitis C virus (HCV) medications to bring about complete cure, reinforces the belief that any viral disease can be effectively treated by specific, safe, and effective anti-viral drugs. However, unfortunately, at present there is no effective drug to treat SARS-CoV-2 infection or any other CoV. The emergence of COVID-19 highlights the need for novel drug discovery for the treatment CoV infections, in particular SARS-CoV-2. The use of a variety of strategies to detect potential inhibitors of SARS-CoV-2 infection is expected to identify a large panel of potential anti-viral compounds. For example, the RNA-dependent RNA-polymerase (RdRp) encoded in the genome of all coronaviruses is a promising candidate for targeting SARS-CoV-2. Given the conserved nature of RdRp among different coronaviruses, drugs that target this protein could constitute a pan-CoV anti-viral drug. However, like with any new drug, development and evaluation processes take time. Therefore, screening of previously approved drugs for anti-viral effects and repurposing them to treat the current pandemic may save time in the development process. Finally, evaluation of the effectiveness of type I interferon combinations, which is known to induce cellular anti-viral responses and to enhance host immunity, in combination with other already known and new drugs, is warranted.

## PREVENTION AND VACCINE

Vaccines against viral diseases have been in use for hundreds of years and have proven powerful in preventing infection. The objective of the vaccines is to generate an effective and safe immune response that will then protect from natural infection. Vaccination may be mediated through passive immunization, which involves injection of components of the immune response, or through active immunization, which triggers an active host response.

Passive immunization provides immediate protection as it does not require a response by the patient, but it does not induce immune memory. In the context of an acute viral infection, such treatment involves administration of anti-viral antibodies that were either derived from the plasma of individuals who recovered from infection or synthetically designed. Treatment can be provided during infection or prophylactically to protect individuals at high risk such as healthcare personnel. It can also be used for prevention following suspected exposure. Transfusion of convalescent plasma was proven effective in treating several viral infections including the H5N1^39^ and H1N1^40^ strains of influenza virus and SARS-CoV,^41^ and treatment protocols were established for Ebola virus^42^,^43^ and MERS-CoV^44^ infections. Accordingly, transfusion of convalescent plasma is likely to be beneficial to SARS-CoV-2,^45^,^46^ yet its effect on virus shedding and disease outcome must be evaluated when given to healthy individuals and patients at different stages and severity of the disease. Importantly, diverse antibodies are produced following viral infection, yet only a fraction of these antibodies is capable of neutralizing the infection. Accordingly, while convalescent plasma could be useful in the short term, it is preferable to identify the most potent antibodies to allow large-scale manufacturing and standardization. As the main target of coronaviruses neutralizing antibodies is the spike protein, samples from patients are screened to identify B cells that produce SARS-CoV-2 antibodies specifically directed at spike. The genes encoding these antibodies are then cloned, and the antigen-binding motif may be used for therapy.

Active vaccines employ attenuated live viruses, inactivated viruses, recombinant viruses, recombinant or purified viral proteins, or virion-like particles that mimic infectious virions. In all cases, the vaccine induces an immune response that should protect against future exposure to the virulent pathogen. When developing a vaccine, it is important to ensure that the vaccine is safe and does not cause major side effects or other unwanted effects that may, in some cases, even exacerbate infection. After the safety tests, vaccine efficacy, which measures how well it protects from disease, is tested. Usually the first test experiments are done in animal models and only then in humans. They involve vaccination and subsequent evaluation of neutralizing antibody production and other potential immune responses. In certain cases, they involve challenge with the virus and long-term follow-up of vaccinated individuals as compared to control groups. The response to immunization must be evaluated in various populations including the elderly who are more vulnerable to infection but unfortunately mount lower responses to vaccines. An effective vaccine is one that induces an immune response that provides protection against infection and pathogen-related disease. Ideally, a vaccine should provide life-long protection, yet short-term vaccines may also be useful, at least during an intermediate period until alternatives are available.

A race to generate a SARS-CoV-2 vaccine is ongoing, involving dozens of candidates in the development pipeline. Among others, new approaches involving RNA and DNA vaccines that enable production of viral antigens upon injection are also being evaluated. A universal coronavirus vaccine designed to target conserved viral epitopes, most likely in the spike proteins, may enable protection from a wide range of related viruses, including CoV types that could emerge in the future. Realistically, vaccination is a long-term goal that could take more than a year. In addition, development of a SARS-CoV-2 vaccine may prove difficult or even unfeasible as is the case for HIV and HCV, which, even after many years of research and huge development budget, are yet to have an effective and safe vaccine. Furthermore, even once a safe and effective vaccine is developed, billions of doses have to be manufactured and distributed worldwide.

## TECHNOLOGIES

The urgent need to control the spread of the SARS-CoV-2 outbreak has brought together a large number of scientists and entrepreneurs to look for solutions that would delay and prevent infection. These solutions include active face masks embedded with anti-viral molecules or particles, fabrics containing anti-viral compounds, anti-viral coatings for surfaces, and various low-toxicity environmental disinfectants. At the same time, ventilators and supportive equipment for patients have been recently developed. Artificial intelligence, Big Data, and machine learning have also been instrumental in tracking virus spread, understanding the epidemiology of the disease, monitoring patients, and preventing the spread of the virus, as well as identifying other clues that may help understand viral pathogenesis towards mitigating and combating COVID-19. Finally, by combining large-scale knowledge, literature, transcriptome data including single-cell RNA sequencing, data learning can help finding potential drug and vaccine targets.

## CONCLUDING REMARKS

Current management of COVID-19 has been mainly dependent on the prevention of infection, including case diagnosis followed by epidemiologic investigation and quarantine, as well as supportive care. Collaborations are key for fighting this new pandemic; it is important both for knowledge acquisition and innovative initiatives for prevention and treatment, but also essential in the modern, globally interconnected world. Lessons learned from other viruses as well as newly collected data and its rapid dissemination, describing virus transmission, genetic evolution, receptor binding, immune response, and pathogenesis, can be combined towards solving the puzzling outbreak and disease. In the battle against the COVID-19 outbreak, virology serves as a major axis around which a variety of other fields of expertise, such as epidemiology, medicine, immunology, mathematics, chemistry, computer and data science, move together.

A key question is whether SARS-CoV-2 will stay with us and become endemic, like the influenza virus and other hCoVs such as hCoV-OC43 and hCoV-229E, or will eventually disappear like SARS-CoV. Endemic viruses may acquire mutations that reduce their virulence, yet the opposite scenario, the emergence of a more dangerous strain, is also possible. Therefore, constant monitoring of the population to examine the incidence of infection as well as the genetic shift of the virus is essential. Furthermore, given the history of coronaviruses, they should be regarded as an ongoing threat to human health, as additional animal viruses of this family may undergo genetic changes enabling them to infect humans. Whether infection with one virus family member confers a certain level of immunity to others, and how long the immunity to a given virus persists, remains to be defined.

Scientists need to know how the virus functions upon infection. This involves virus cultivation and propagation in cell culture, and development of appropriate cell/organ culture and animal models. Laboratory studies should also describe the virus host range, which is primarily determined by the susceptibility or permissiveness of different cells. Further basic study of the biology of these viruses is warranted as it may identify new targets for therapy.

## Abbreviations

CoV: coronavirus
COVID-19: coronavirus disease 2019
hCoV: human coronavirus
SARS: severe acute respiratory syndrome.
